# Choroid Plexitis and Obstructive Hydrocephalus Secondary to Cryptococcal Meningoencephalitis in an Immunocompetent Individual With Remarkable Imaging Findings: A Case Report

**DOI:** 10.7759/cureus.65544

**Published:** 2024-07-27

**Authors:** Antonio Santana Veliz, Nneka F Onwumere, Amar Swarnkar, Rajiv Mangla

**Affiliations:** 1 Diagnostic Radiology, Upstate University Hospital, Syracuse, USA; 2 Neurosurgery, Upstate University Hospital, Syracuse, USA; 3 Neuroradiology, State University of New York Upstate Medical University, Syracuse, USA

**Keywords:** choroid plexitis hydrocephalus case, choroid plexitis in cryptococcosis, immunocompetent cryptococcal meningitis, cryptococcal meningitis neuroimaging, mri findings choroid plexitis, neuroimaging cryptococcal infection, choroid plexitis imaging, fungal infection in immunocompetent, cryptococcal meningoencephalitis, choroid plexitis

## Abstract

*Cryptococcus* is an invasive and opportunistic fungus usually associated with immunocompromised individuals. Invasion of the choroid plexus by *Cryptococcus* is rare. This report presents the radiologic findings of a previously healthy male with bilateral choroid plexus invasion complicated by obstructive hydrocephalus.

## Introduction

*Cryptococcus neoformans* is a fungus capable of crossing the blood-brain barrier and invading the CNS. Upon invasion, it can cause intracranial inflammation, manifesting as meningoencephalitis, encephalitis, meningitis, or ventriculitis [1]. The infection usually starts in the respiratory system, colonizing the lungs, and then spreads hematogenously to the brain. Inflammation of the choroid plexus due to a fungal infection is rarely reported. Although typically an opportunistic infection, we present an unusual case involving an immunocompetent individual with no identifiable risk factors. This report details the patient’s history, clinical evaluation, and management.

## Case presentation

A 56-year-old male with no significant past medical history presented to the emergency department with a two-month history of difficulty eating, nausea, and blurred vision often associated with headaches. He also had a one-month history of memory loss, unintentional weight loss, gait instability, intermittent back pain, and flank pain with dark urine. Additionally, the patient reported an upper respiratory tract infection three months prior to his presentation. He initially left the emergency department without being seen but returned one month later via emergency medical services for worsening dizziness, episodes of nausea and vomiting, profound confusion, hallucinations, and an inability to walk.

**Figure 1:**
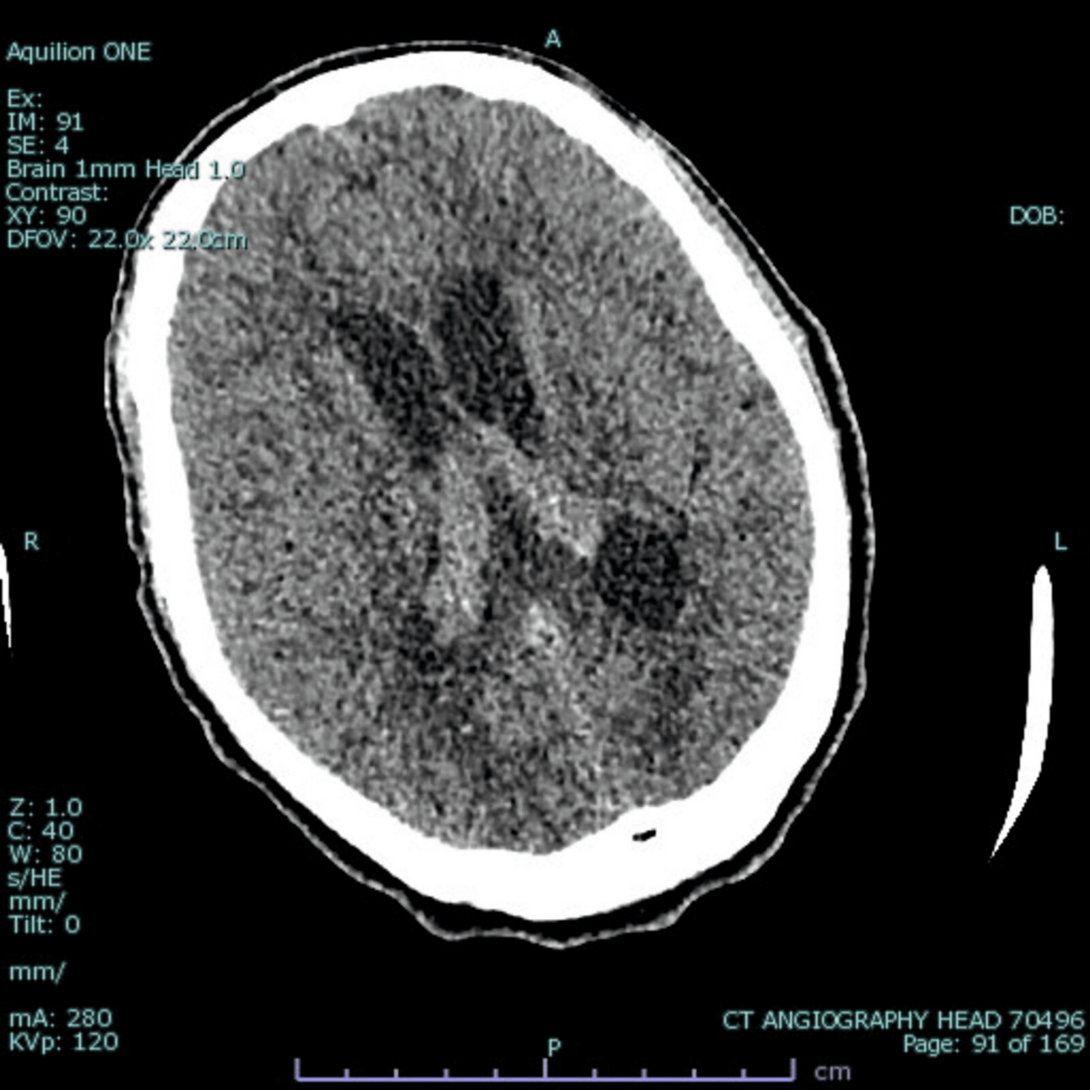
Non-contrast CT of the head showing prominent bilateral choroid plexuses and hydrocephalus affecting the bilateral temporal horns, with surrounding hypodensity

**Figure 2:**
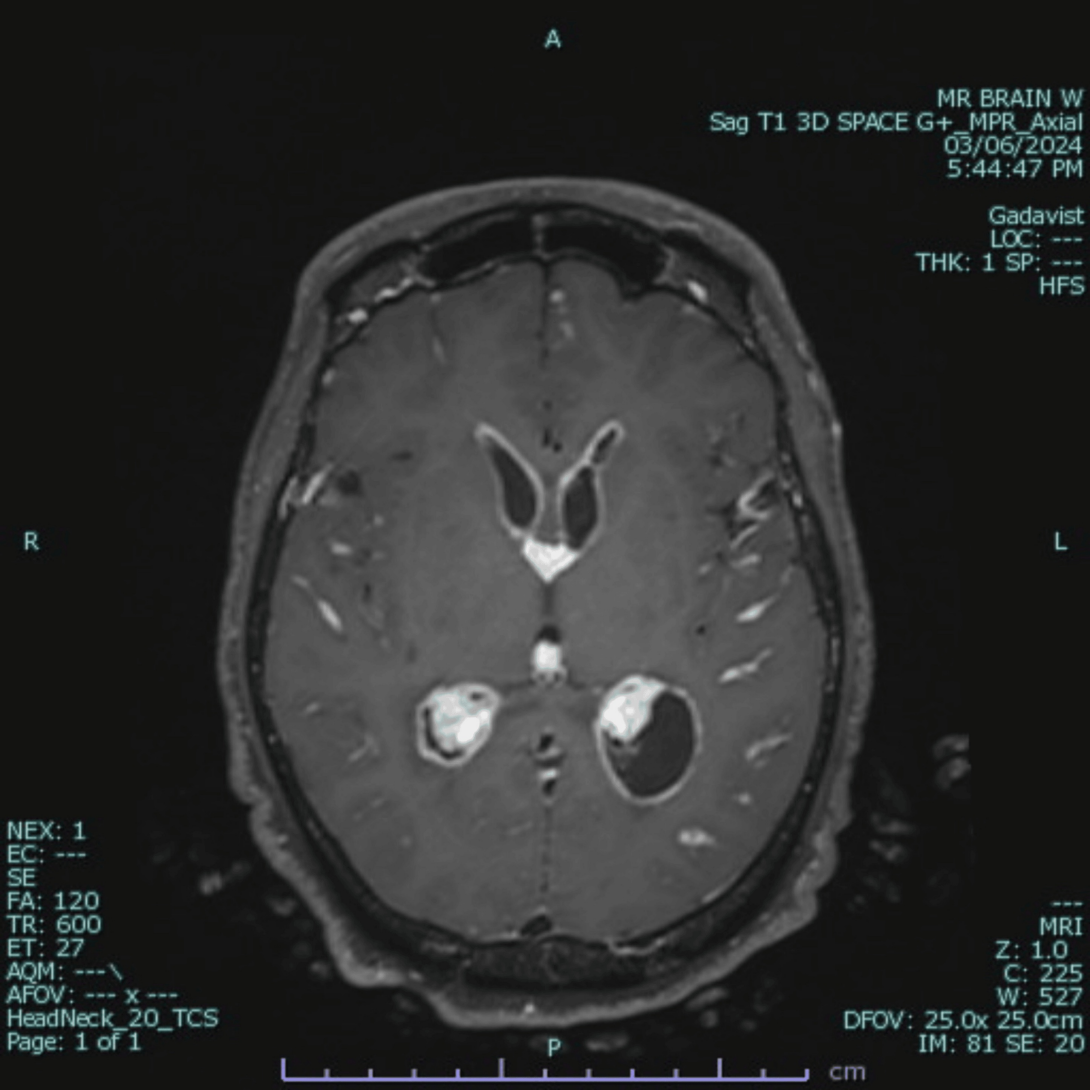
Axial T1-weighted post-contrast MRI of the brain demonstrating leptomeningeal enhancement of cranial vessels, ventricles, pia mater, and the choroid plexus

**Figure 3:**
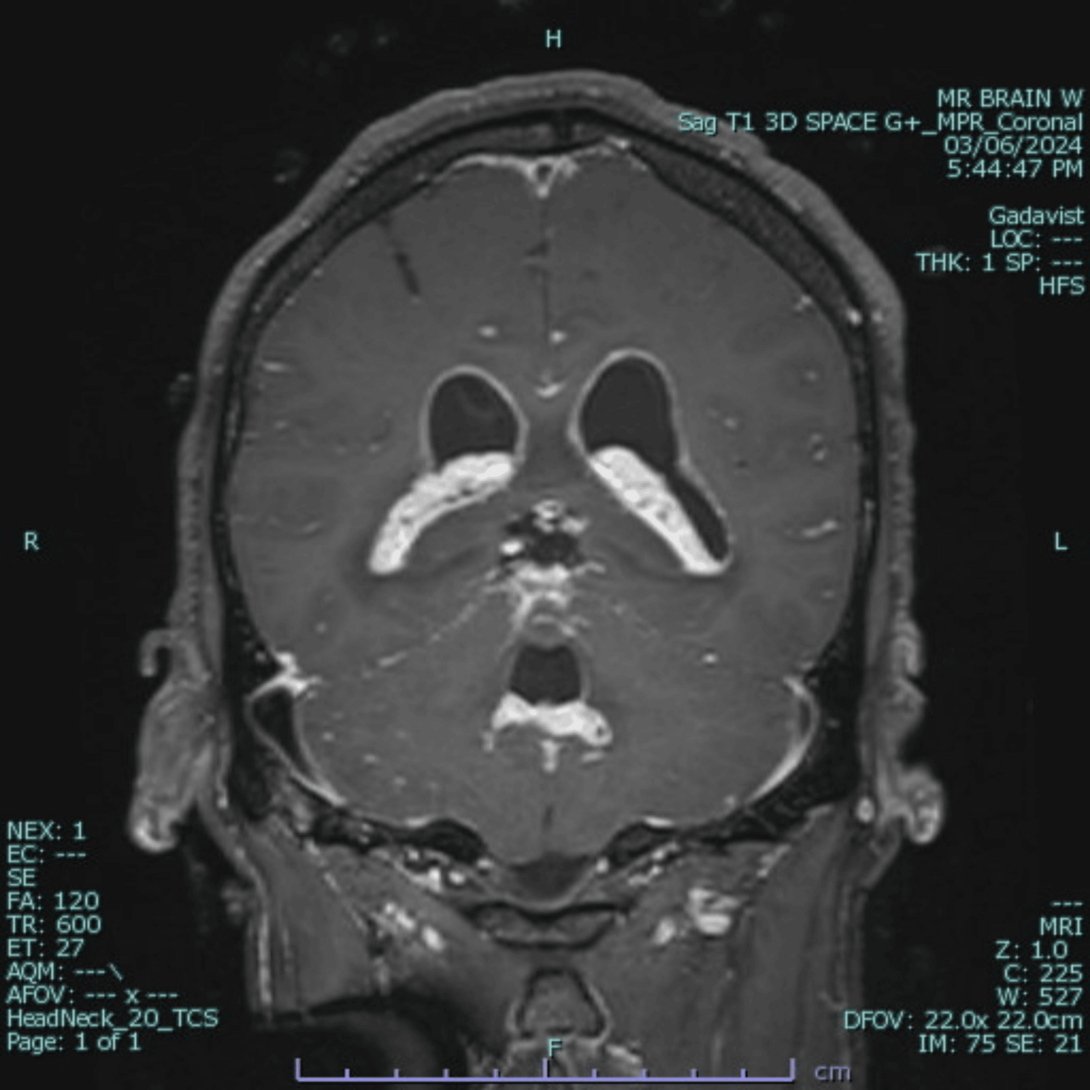
Coronal T1-weighted post-contrast MRI showing ventriculomegaly, leptomeningeal enhancement of the pia mater and choroid plexus extending to the foramina of Magendie and Luschka, and edema of the cerebellar dentate nucleus

**Figure 4:**
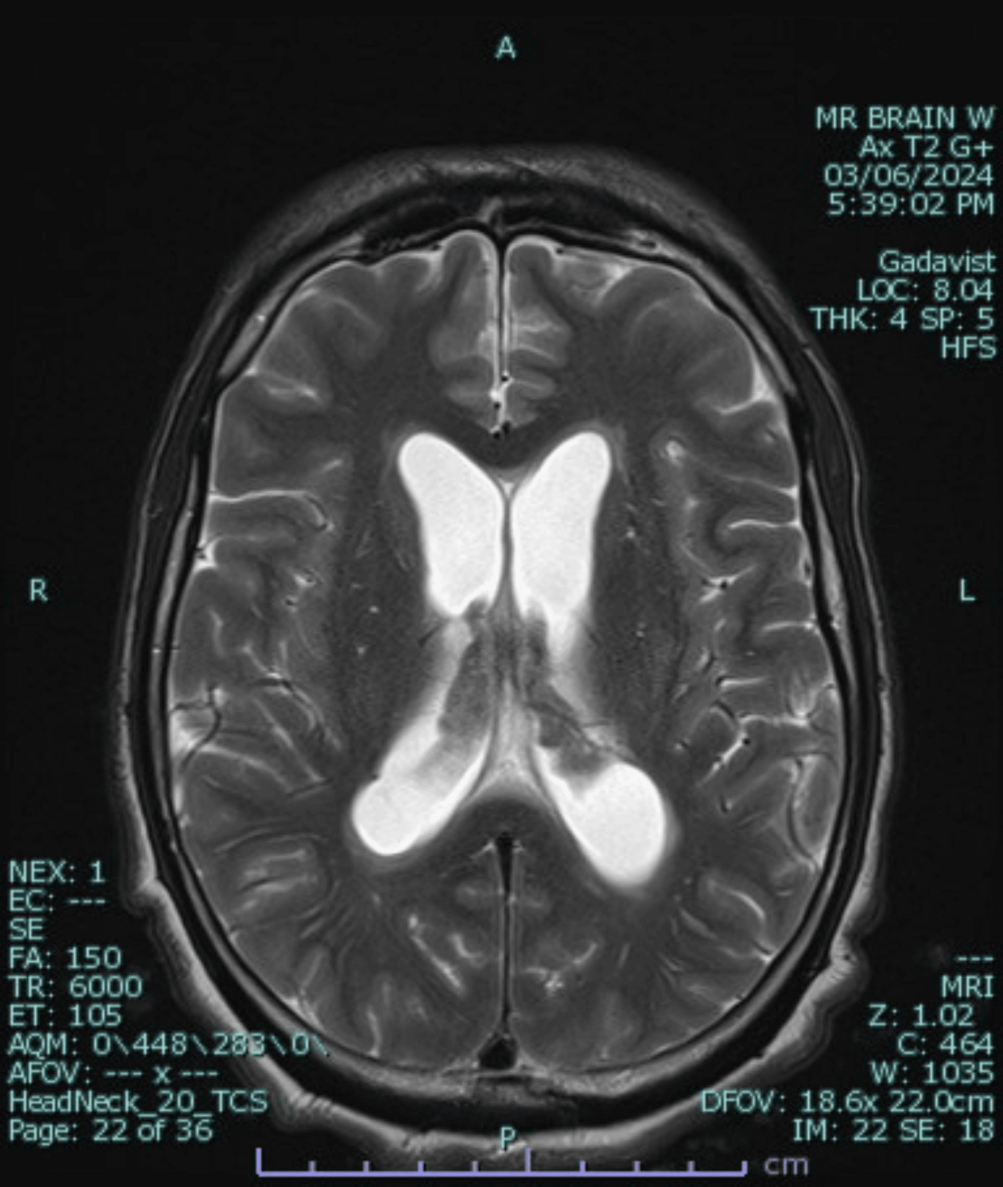
Axial T2-weighted MRI showing prominent bilateral hydrocephalus, choroid plexitis, and periventricular white matter edema

The patient was admitted to the intensive care unit for further management. Clinical, laboratory, and radiological findings collectively established a diagnosis of choroid plexitis secondary to cryptococcal meningoencephalitis. CSF results revealed positive cryptococcal antigen, pleocytosis, elevated protein levels, and low glucose levels. An infectious disease specialist was consulted, and HIV and CD4 count tests returned negative results for the patient. CT imaging showed low-lying cerebellar tonsils, hydrocephalus with bilateral temporal horn enlargement, and involvement of the fourth ventricle (Figure 1). MRI revealed leptomeningeal and ependymal enhancement in all ventricles, the foramen of Monro, and the foramina of Luschka, as well as hyperdense choroid plexuses (Figures 2-4).

The patient initially required external ventricular drain (EVD) placement to manage ventriculomegaly and control intracranial pressure. The EVD was removed once the hydrocephalus resolved and imaging showed adequate ventricular decompression. He was treated with intravenous antifungals, including liposomal amphotericin B and flucytosine, over six weeks, leading to clinical improvement. Repeated CSF examinations demonstrated reductions in total protein and cell counts, while metabolic panels indicated stable electrolyte levels. After the induction therapy, the patient underwent eight weeks of consolidation therapy with high-dose oral fluconazole (800 mg) and prednisone. Elevated transaminases necessitated a reduction in fluconazole dosage to 400 mg daily. During his hospital stay, the patient received speech-language therapy for communication and swallowing rehabilitation, as well as physical and occupational therapies for balance, strength, and functional mobility. Following consolidation therapy, he continued maintenance therapy with oral fluconazole (200 mg daily for 12 months) and had tri-weekly lab tests to monitor liver function. Although he reported persistent double vision and occasional headaches post-discharge, these symptoms remained stable throughout his treatment.

## Discussion

*C. neoformans* is classified as an encapsulated yeast-like fungus that generally provokes clinical manifestations in immunocompromised individuals. These include organ transplant recipients, individuals on immunosuppressive therapy, and those with HIV/AIDS. The most common forms of exposure are from soil contaminated by bird excrement and decaying wood [1]. It is one of the very few fungal species that has acquired the ability to cross the blood-brain barrier and infect the CNS [2]. Infection occurs via inhalation, most commonly affecting the lungs and causing pneumonia-like symptoms, but it will invade the CNS via hematogenous spread. The polysaccharide capsule of the fungus inhibits phagocytosis by impairing neutrophil migration to the infection site, facilitating optimal infectivity [3]. Clinical features of cryptococcal infection can include headache, fever, nausea, vomiting, sensitivity to light, confusion, and changes in mental status. Diagnosis of cryptococcal infections typically involves laboratory tests, such as culturing the fungus from bodily fluids or tissues or detecting its components in CSF for CNS infections.

The range of radiological findings includes dilated perivascular spaces, gelatinous pseudocysts, intraparenchymal cryptococcomas, miliary nodules, meningeal involvement, intraventricular/choroid plexus enhancement, and hydrocephalus. Dilated Virchow-Robin (VR) spaces in the basal ganglia are reported as the most common imaging finding [4].

These imaging findings differ between immunocompetent and immunodeficient individuals. In immunocompetent individuals, MRI typically shows intraparenchymal cryptococcomas and enhancement. Conversely, in immunodeficient individuals, MRI can be normal or show mildly dilated VR spaces, cortical atrophy, and rarely, meningeal enhancement [5].

In our case, significant imaging findings included choroid plexitis with extensive leptomeningeal enhancement involving the cranial vessels, ventricles, and pia surface. There was also severe ependymal enhancement involving all the ventricles and choroid plexus, extending to the level of the foramina of Magendie and Luschka, resulting in obstructive hydrocephalus and ventriculomegaly.

Choroid plexitis is a condition involving inflammation of the choroid plexus, a structure within the ventricles of the brain responsible for producing CSF that provides cushioning support for the brain and spinal cord. Kumari et al. suggest the choroid plexus is an alluring target for CNS entry by various microorganisms due to its location at the interface between the CSF and systemic circulation [6]. Choroid plexitis can have various causes, including infections (such as bacterial, viral, or fungal meningitis), autoimmune conditions, trauma, or tumors affecting the choroid plexus. Inflammation of the choroid plexus can lead to increased production of CSF, potentially resulting in hydrocephalus and increased pressure within the skull. This increased pressure can cause symptoms such as headaches, nausea, vomiting, and changes in vision or mental status. Treatment involves addressing the underlying cause of inflammation. This may include antibiotics or antiviral or antifungal medications for infectious causes, immunosuppressive therapy for autoimmune conditions, or surgical intervention to relieve hydrocephalus in severe cases. Management of symptoms, such as controlling intracranial pressure, may also be necessary to alleviate discomfort and prevent complications.

Treatment for CNS cryptococcosis usually involves antifungal medications, such as amphotericin B and fluconazole. The duration and specific regimen of treatment may vary depending on the severity of the infection and the patient’s overall health, but maintenance therapy is recommended with fluconazole after eight weeks of induction and consolidation phases [7].

## Conclusions

The presence of imaging findings such as a strongly enhancing choroid plexus, cystic lesions in the basal ganglia, meningeal/choroid plexus enhancement, hydrocephalus, and intraventricular lesions in an immunocompetent patient presenting with headache, vomiting, confusion, hallucinations, and an inability to walk should suggest a diagnosis of cryptococcal infection. This case underscores the importance of considering cryptococcal infection even in immunocompetent individuals when these specific imaging and clinical features are observed.
